# Oxidation and metal-insertion in molybdenite surfaces: evaluation of charge-transfer mechanisms and dynamics

**DOI:** 10.1186/1467-4866-9-8

**Published:** 2008-06-05

**Authors:** CV Ramana, U Becker, V Shutthanandan, CM Julien

**Affiliations:** 1 Nanoscience and Surface Chemistry Laboratory, Department of Geological Sciences, University of Michigan, Ann Arbor, Michigan 48109, USA; 2 Department of Metallurgical and Materials Engineering, University of Texas at El Paso, El Paso, Texas 79968, USA; 3 Environmental Molecular Sciences Laboratory, Pacific Northwest National Laboratory, Richland, WA 99352, USA; 4 Institut des Nano-Sciences de Paris, CNRS-UMR 7588, Université Pierre et Marie Curie Campus Boucicaut, 140 rue de Lourmel, 75015 Paris, France

## Abstract

Molybdenum disulfide (MoS_2_), a layered transition-metal dichalcogenide, has been of special importance to the research community of geochemistry, materials and environmental chemistry, and geotechnical engineering. Understanding the oxidation behavior and charge-transfer mechanisms in MoS_2 _is important to gain better insight into the degradation of this mineral in the environment. In addition, understanding the insertion of metals into molybdenite and evaluation of charge-transfer mechanism and dynamics is important to utilize these minerals in technological applications. Furthermore, a detailed investigation of thermal oxidation behavior and metal-insertion will provide a basis to further explore and model the mechanism of adsorption of metal ions onto geomedia.

The present work was performed to understand thermal oxidation and metal-insertion processes of molybdenite surfaces. The analysis was performed using atomic force microscopy (AFM), scanning electron microscopy (SEM), transmission electron microscopy (TEM), Rutherford backscattering spectrometry (RBS), and nuclear reaction analysis (NRA).

Structural studies using SEM and TEM indicate the local-disordering of the structure as a result of charge-transfer process between the inserted lithium and the molybdenite layer. Selected area electron diffraction measurements indicate the large variations in the diffusivity of lithium confirming that the charge-transfer is different along and perpendicular to the layers in molybdenite. Thermal heating of molybenite surface in air at 400°C induces surface oxidation, which is slow during the first hour of heating and then increases significantly. The SEM results indicate that the crystals formed on the molybdenite surface as a result of thermal oxidation exhibit regular thin-elongated shape. The average size and density of the crystals on the surface is dependent on the time of annealing; smaller size and high density during the first one-hour and significant increase in size associated with a decrease in density with further annealing.

## Background

Sulfide minerals and the associated geological/physical/chemical processes are an active research topic for mineralogists, geochemists, and geotechnical/environmental engineers. Sulfide ores constitute a major source of metals, especially noble metals. In addition, the rich diversity in crystal chemistry, surface reactivity, phase transformations, stability, thermodynamics, and electronic properties makes the sulfide minerals attractive for a wide variety of industrial applications, such as lubricants and catalysts [1,2]. Molybdenite (MoS_2_) belongs to the family of the transition-metal dichalcogenide (TMD) minerals with the formula *MX*_2 _(where M = Cd, Ti, Mo, Sn and X = I, S, Se). Due to their layered structure, TMDs are often referred to as two-dimensional (2D) solids. The 2D structure of these minerals is due to the strong covalent or ionic bonding within a layer while individual layers are held together by weaker van-der-Waals forces. Even though the latter are often referred to as "van der Waals" type of interactions, some contributions from covalent and ionic interactions are also possible, particularly in the metal inserted complexes [3,4]. These compounds exhibit anisotropic physical properties, such as different conductivity parallel and perpendicular to the layers, ranking MoS_2 _the most anisotropic 2D material after graphite [3].

Molybdenite is the essential ore mineral of the molybdenum industry for production of Mo metal and Mo-based compounds, such as sodium and calcium molybdates, ammonium paramolybdate, and molybdenum trioxide [5]. Molybdenite-based formulations are extensively used in industrial machinery and weapons for lubrication [6]. Molybdenite is a widely used catalyst in CO hydrogenation and hydrodesulfurization processes for the production of cleaner fuels [7,8]. Mo is a redox-sensitive trace metal and becomes enriched in sulfidic, reducing, and organic rich sediments. Molybdenite is found in deep-sealed veins associated with scheelite, wolframite, topaz, and fluorite [9].

**Figure 1:**
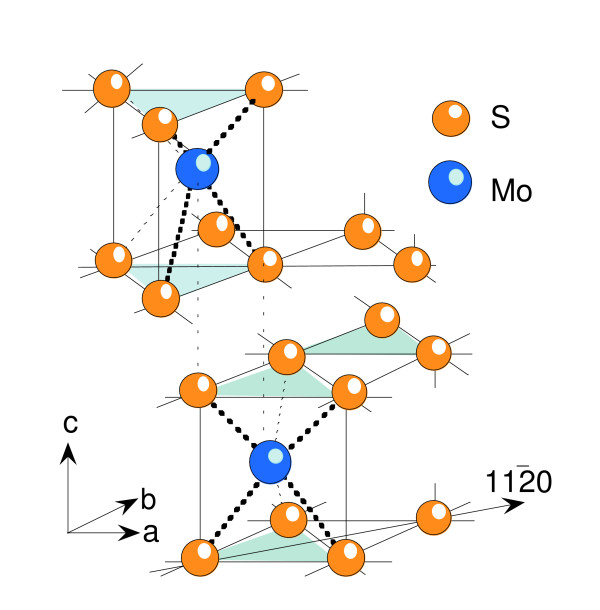
Perspective view of the structure of 2*H*_*b*_-MoS_2_. The crystallographic axes for the *H *structure are those for standard hexagonal systems with *a*(*x*,*y*) parallel and *c*(*z*) perpendicular to the layers. The coordination of Mo atoms are trigonal prismatic with the stacking sequence *AbA-BaB *along the *c *direction.

The mineral molybdenite belongs to the group VI TMD compounds that adopt the 2*H*_*b *_polytype, in which the metal atoms are staggered. The perspective view of the structure of MoS_2 _is shown in Fig. 1. The hexagonal phase 2*H*-MoS_2_, also denoted as β-MoS_2 _[10], crystallizes with *P6_3_/mmc *($\equiv D_{6h}^{4}$) symmetry with lattice parameters *a *= 3.161(1) Å and *c *= 12.295(2) Å (the numbers in the parentheses represent the error). MoS_2 _has perfect (001) cleavage and a density of 4.62–4.73 g/cm^3 ^[9]. Naturally occurring molybdenite has been found to exist mostly in 2*H *stacking polytypes but 3*R *ones are also available.

The objective of the present work is to understand the effects of thermally-induced oxidation and alkali metal (Li) ion insertion in molybdenite surfaces. Understanding the oxidation behavior of MoS_2 _is crucial because several applications of MoS_2 _are influenced by its degree of oxidation. Similarly, information on the mechanism of foreign metal-atom insertion into the molybdenite structure is important to understand the associated electronics structure changes. Therefore, the present investigation was made using a wide variety of analytical techniques such as atomic force microscopy (AFM), scanning electron microscopy (SEM), transmission electron microscopy (TEM), Raman spectroscopy (RS), Rutherford backscattering spectrometry (RBS), and nuclear reaction analysis (NRA) to elucidate how the oxidation proceeds in these minerals at higher temperatures. In addition, the structural effects associated with metal insertion at room temperature were evaluated. Our choice of lithium (Li) was due to the following reasons: (1) The chemistry of Li, being a simple metal, is well known, (2) the metal insertion in molybdenite is only possible with small, strongly reducing guests, such as the alkali metals [3,10] and (3) comparison of the results will be made easy in view of several existing reports on Li in synthetic molybdenite [3,10]. Using the familiar surface analytical techniques, namely AFM, SEM and TEM, is to probe the changes in surface structure and morphological features due to thermal oxidation and metal-ion insertion in molybdenite. The combined use of RS and TEM measurements allow probing the information on the local chemical structure and bonding and are quite useful to evaluate the impact of metal-ions on the local structural environment in molybdenite. Using the less familiar ion-beam analytical techniques, such and NRA, is mainly to understand the thermal oxidation behavior of molybdenite. High-energy ion-beam analysis using nuclear reactions, particularly NRA measurements, provide an important tool to measure the absolute concentration of lighter elements, such as hydrogen (H), carbon (C), nitrogen (N), and oxygen (O), and makes it quite useful to understand the science and processes at geochemical media [11-13]. Therefore, we have used the NRA to probe the surface oxide growth on molybdenite surface.

### Experimental

Two sets of naturally-occurring molybdenite samples were used in this work. The minerals, found in the mineral storage/collection at the Department of Geological Sciences at the University of Michigan, of unknown origin were employed for thermal oxidation experiments. Molybdenite samples obtained from Japan were employed for lithium-insertion measurements. Atomic force microscopy measurements were performed using a Digital Instruments AFM (NanoScope IV). The measurements were made in tapping mode. Scanning electron microscopy observations were made using two different instruments. A JEOL (model 6150) electron microscope was used to image the oxidation behavior of the molybdenite surfaces as a function of heating time. SEM imaging experiments on lithium-reacted molybdenite samples were made using a high-resolution electron microscope (Hitachi S-4700). Raman scattering (RS) spectra were measured using a Jobin-Yvon U1000 double-pass spectrometer equipped with a cooled, low-noise photomultiplier tube (ITT FW130). The incident light used for the experiments was the 515 nm Ar ion laser. TEM analysis was performed using a JEOL TEM 2010F at a 200 kV acceleration voltage. Phase and structure of the material were monitored using selected area electron diffraction (SAED). Specimens for TEM analysis were prepared by dispersing the MoS_2 _sample on 3-mm Cu grid with a hole size of 1 × 2 mm. High resolution transmission electron microscopy (HRTEM) image processing including the fast Fourier transformation (FFT) was carried out using a Gatan Digital Micrograph 3.4.

Lithium intercalated Li_x_MoS_2 _samples ware prepared from natural 2*H*-MoS_2 _treated with dilute butyllithium in hexane in a controlled water and oxygen-free environment. Prolonged treatment (~10 days) with very dilute solutions (~0.005 *M*) was used to produce the lithium-inserted samples for analytical experiments. On cleaving the surface was seen to be broken into mm sized regions of crystal, either shiny, or matt black (poor surface quality) indicating the intercalated areas.

Thermal oxidation experiments were performed in air. Molybdenite surfaces were heated to 400°C in a furnace. Various analytical measurements were performed as a function of heating time to understand the thermally-induced effects. The ion-beam experiments namely Rutherford backscattering spectrometry (RBS) and nuclear reaction analysis (NRA) measurements were carried out in the accelerator facility at the Environmental Molecular Sciences Laboratory (EMSL) of the Pacific Northwest National Laboratory (PNNL), Richland, Washington, USA. A helium (He^+^) ion beam of 2 MeV was incident on the sample surface at near normal and the scattered ions were detected at an angle of 170° from the sample normal for RBS measurements. A beam of 0.94 MeV deuterium (d^+^) ions was incident on the molybdenite sample and the reaction products were detected at a scattering angle of 170° from the sample normal for NRA measurements. A thin aluminized mylar film covered the detector to stop backscattered deuterium ions, allowing only the more energetic reaction products to enter the detector. The detected particles for these measurements were protons from the ^16^O(d,p)^17^O reaction.

## Results and Discussion

### Pristine molybdenite surfaces

**Figure 2:**
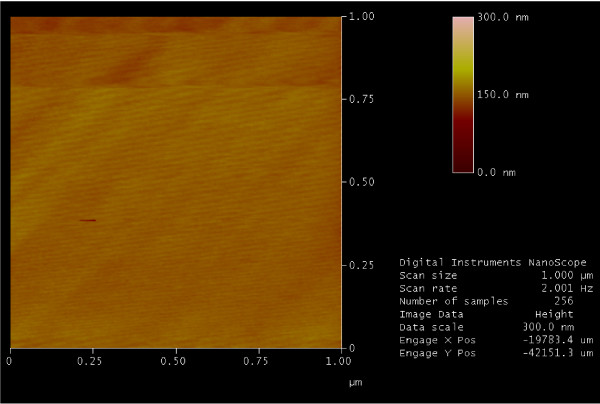
AFM image of the molybdenite surface.

**Figure 3:**
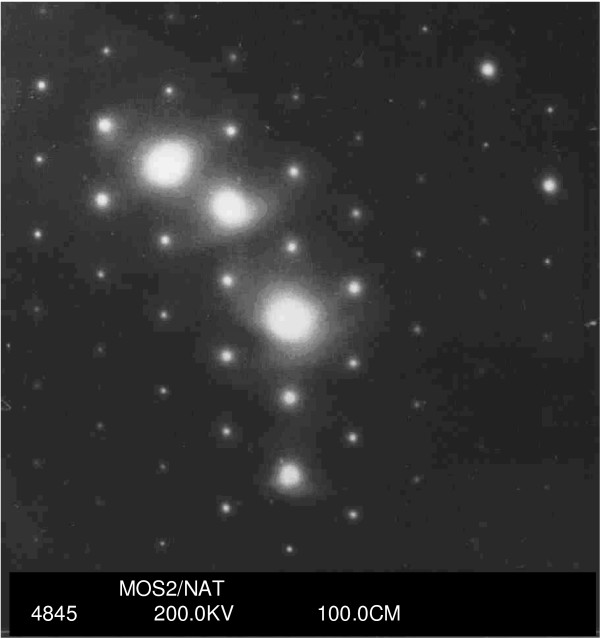
Electron diffraction diagram of molybdenite (2*H*-MoS_2 _polytype) showing the hexagonal lattice.

The AFM image of a representative pristine natural MoS_2 _sample surface is shown in Fig. 2. Molybdenite surfaces are often atomically flat over relatively large areas as seen in this work (Fig. 2) and other reports [14-16]. AFM images, in the area of scanning, show no specific surface features such as steps or kinks. The electron diffraction pattern of the molybdenite crystal is shown in (Fig. 3). The hexagonal lattice of the 2*H *polytype is evident from the image (Fig. 3).

**Figure 4:**
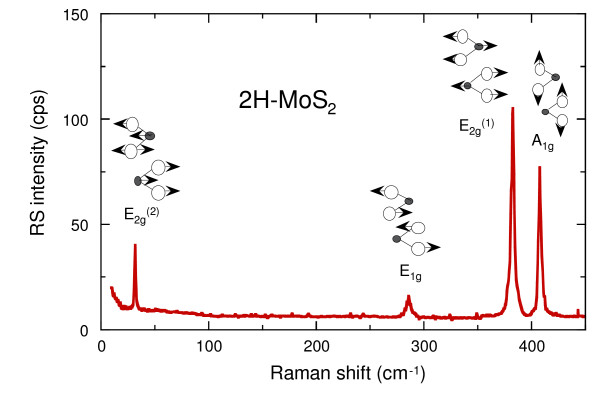
Raman scattering spectrum of 2*H*-MoS_2 _single crystal showing the anisotropic character of the vibrational modes. The interlayer shear mode observed at 33.5 cm^-1 ^is typical of the trigonal prismatic structure.

The long-wavelength lattice vibrations of molybdenite are interesting and often provide a model system to understand the linear-chain model of the layered-type chalcogenide minerals. Group theory predicts two infrared (IR) and four Raman-active modes for 2H-MoS_2 _[16-18]. The Raman scattering spectrum of the pristine molybdenite surface recorded at ambient temperature is shown in Fig. 4 along with the associated mode assignments. The *A*_1*g *_mode at 407 cm^-1 ^is an intralayer mode involving the motion of S atoms along the c axis. The *E*_*g *_mode at 383 cm^-1 ^is an intralayer vibrational mode involving motion of Mo+S atoms in the basal plane. The peak at 286 cm^-1 ^with weak intensity and *E*_1*g *_symmetry is observed, which involves S atoms in the basal plane. The spectrum of the pure 2*H*-MoS_2 _exhibits a rigid-layer (RL) mode *E*_*g *_at 32 cm^-1^. This mode is of interlayer type involving rigid motion of neighboring sandwiches in anti-phase. In layered crystals, the frequency of the RL mode provides direct information on the strength of the interlayer forces in these crystals, since for such RL motions the restoring forces are provided entirely by layer-layer interactions [19].

### Lithium insertion

**Figure 5:**
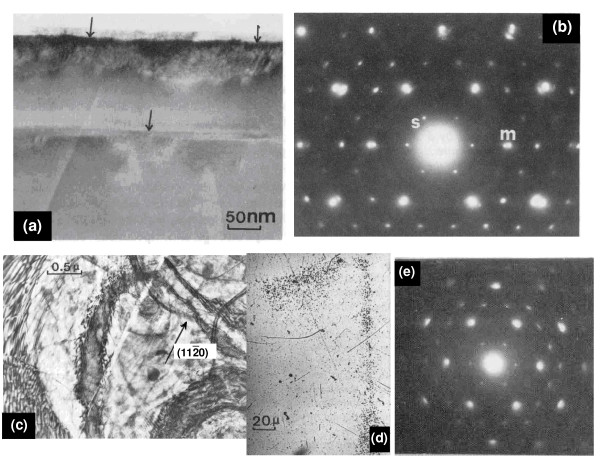
Electron micrographs of Li-inserted MoS_2 _by the n-butyllithium method. (a) After 10 min, defects are created near the edges and in the steps of the specimen denoted by arrows. (b) After 2 h intercalation, superlattice spots appear (denoted by the letter *s*), which are indexed as (1/201/20). Notice also the splitting of the main spots (denoted by the letter m). (c) A micrograph taken from the same area reveals that the specimen is heavily defected owing to intercalation. (d) Distribution of lithium vs. distance from the edges of the specimen as revealed by SSNTD images. (e) Splitting of the ($10\bar{1}0$) spots.

Figure 5 shows the SEM and electron diffraction micrographs of Li-inserted molybdenite produced by inserting the samples in n-butyl-lithium. It can be seen that some defects are created near the edges and in the steps of the specimen, after 0.2 hours of insertion (arrows in Fig. 5a). After 2 hours of insertion reaction, superlattice spots appear (label *s *in Fig. 5b). Notice also the splitting of the main spots denoted by the letter m. The micrograph taken from the same area reveals that the specimen is heavily disordered or contains a significant number of defects as a result of metal-insertion reaction in the molybdenite structure (Fig. 5c). The distribution of lithium vs. distance from the edges of the specimen has been determined by tracer studies using solid-state nuclear track detectors (SSNTD). A typical SSNTD image of Li_0.3_MoS_2 _is also shown in Fig. 5 (Fig. 5d). Continued insertion reaction for 20 hours results in the appearance of superlattice spots in areas far from the edges. Splitting of the ($10\bar{1}0$) spots still occurs, the inner spots being very intense while the outer ones are faint as shown in Fig. 5 (Fig. 5e). This is an indication that the intercalated system did not reach saturation, even locally, although the diffusion of lithium has progressed, implying large variations in the diffusivity of lithium in molybdenite.

**Figure 6:**
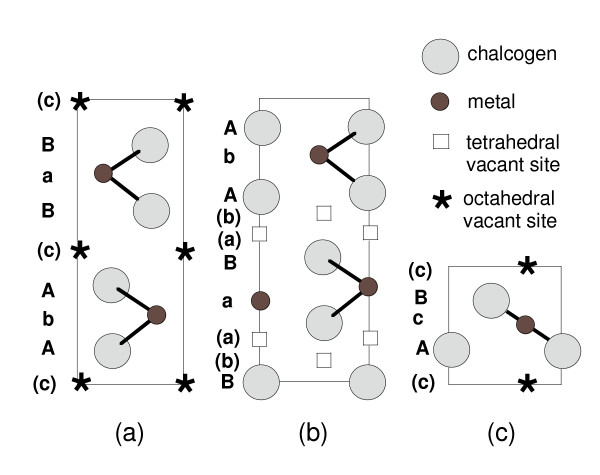
Schematic representation of the *MX*_2 _layered structure showing the empty sites available for Li insertion. (a)The (1120) projection of the unit cells of 2*H*_*a*_-*MX*_2 _with tetrahedral inter-sandwich sites, (b) 2*H*_*b*_*-MX*_2 _with octahedral inter-sandwich sites and (c) the 1*T*-Li*MX*_2 _structure.

The results of the lithium insertion reaction and associated structural modifications are discussed as follows. Insertion of foreign metal and/or ionic species into a host structure implies two main conditions: (1) the structural ability to accept ions in empty sites, and (2) the presence of acceptor levels in the electronic structure. From a crystal structure point of view, there are several possible locations to insert lithium into molybdenite (Fig. 6) as the unit cell of 2*H*_*b*_-*MX*_2 _possesses tetrahedral and octahedral inter-sandwich sites (this is equally applicable for inserting any other metal ions into the structure). Among the group-VI TMDs, MoS_2 _is a typical example where insertion reactions induce a local structure modification. In this particular case, Mo presents a trigonal prismatic (*TP*) S coordination which changes to the octahedral (*Oh*) one, i.e. the *TP *→ *Oh *transition [3,20-22]. The structure modification is accompanied by an increase in the Mo-S bond ionicity in agreement with the respective stability of the new atomic arrangement, the Coulomb repulsion between partially charged ligands favoring the octahedral form. Also, comparison of the *d*-band density of states for 2*H*-MoS_2 _and hypothetical 1*T*-LiMoS_2 _shows that the occupied bands which contain six states are lower in the case of the *Oh *phase corresponding to the glide process between Mo and S atoms. This is a good example of destabilization through lithium reduction. The transformation from *TP *to *Oh *coordination is attributed to a process which is driven by a lowering of the electronic energy for the octahedral structure when the charge transfer occurs from Li ions to the MoS_2 _layer (electrons are donated by Li) upon insertion reaction [3,10].

### Thermal oxidation

**Figure 7:**
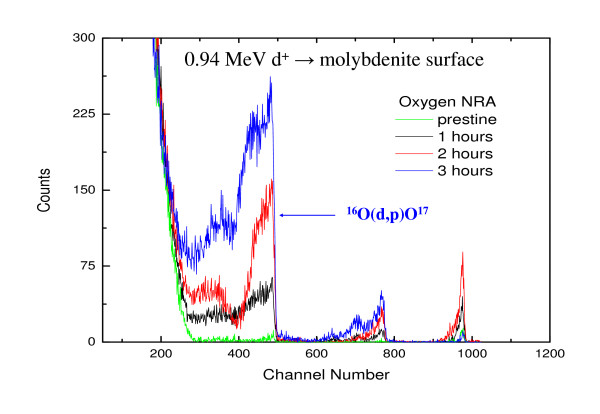
NRA curves of molybdenite surfaces as a function of heating time.

The NRA results are shown in Fig. 7. The curves are shown as a function of heating time of molybdenite surfaces in air. The molybdenite surface, introduced into the high-vacuum chamber immediately after cleavage, did not show any peak corresponding to oxygen. This observation indicates that there is no presence of oxygen on the pristine molybdenite surface before the thermal oxidation experiments. We claim that we start our thermal oxidation experiments with atomically clean molybdenite surfaces since the detection limit of NRA to determine the oxygen concentration (using the ^16^O(d,p)O^17 ^reaction) is ~3 × 10^15 ^atoms/cm^2^. The evolution of the oxygen peak as a function of heating time is evident in the NRA spectra. The peak intensity increases with the increase in time of heating, which indicates progressive thermal oxidation of the molybdenite surfaces. However, the increase in oxygen peak intensity is somewhat low during the first hour of heating. This observation indicates that the oxidation during the first hour of heating is slow leading to oxidation only on the surface. A significant increase in peak intensity and change in shape (becoming more broad) with further heating after the first hour indicates that oxidation is progressively proceeding from the surface layers into the deeper layers.

**Figure 8:**
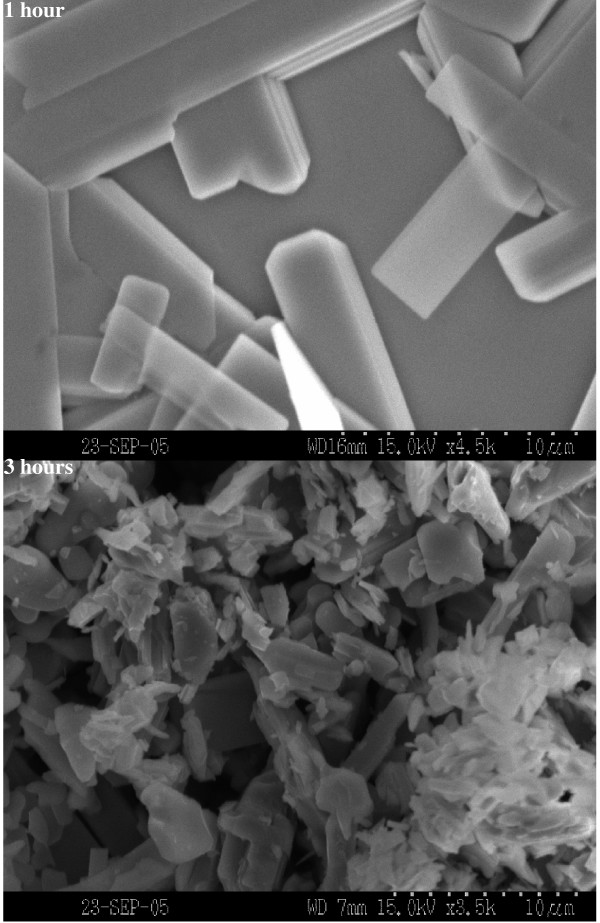
SEM images of molybdenite surfaces as a function of heating time.

SEM micrographs of the molybdenite surfaces are shown in Fig. 8 as a function of heating time. The thermally-induced oxidation completely changes the morphology of the molybdenite surface. The SEM images indicate the formation of small oxide crystals on the surface within the first hour of heating. More significant changes are observed after 2 to 3 hours of heating time. The crystals formed are light greenish indicating the oxide phases. The number of the oxide crystals formed on the surface increases significantly as a result of thermal oxidation. The crystals formed on the molybdenite surface as a result of thermal oxidation are assuming a regular shape with an elongated thin structure, which is in good agreement with the shapes reported for Mo oxides [23]. Oxide crystals formed on the surface as a result thermally-induced oxidation during the first one hour of heating are, however, longer in size extending over 4–5 microns in length and about 2 microns in width. The SEM image shown for samples after 3 hours of heating clearly shows the density of such crystals formed on the surface increases significantly. The increase in the number of crystals at 3 hours of heating increases the surface roughness with random distribution of the crystals in addition to the irregular shapes. However, an interesting phenomenon is the size of the crystals. It can be seen that the crystals formed at 3 hours of heating are smaller in size when compared to the ones formed in the first hour of heating. It may be that the tendency of all crystals to grow within the limited surface area may inhibit the growth of larger crystals in the neighboring medium. The accumulation of several crystals contributing to the rough morphology is also observed in some areas, which supports the idea that the higher density of oxide crystals may inhibit the growth of large and uniform crystals as seen at the initial stages of oxidation. In other words, the formation of continuous oxide layers, initially formed on the top-most surface layers, may influence the lateral diffusion of the particles to join the already formed crystals and to grow into larger crystals.

## Conclusion

The thermally-induced oxidation behavior and lithium metal-insertion reaction effects on molybdenite surfaces are studied using a combination of structural imaging and spectroscopic measurements. Starting from pristine and well-ordered molybdenite layers, local disordering of the structure occurs as a result of charge-transfer process between the inserted metal ions and the molybdenite layer. Heating of the molybdenite surface in air at 400°C induces oxidation. Oxidation in the first hour of heating is initially slow and then increases significantly.

## Authors' contributions

CVR carried out sample collection, performed the measurements of SEM, AFM and NRA on the prestine and oxidized MoS_2 _samples, interpreted data, and drafted the manuscript, UB is involved in planning, coordination and support for all the measurements and assisted with data interpretation and drafting the manuscript, VS assisted with the preparation of samples, experiments and data collection, and interpretation of NRA results obtained, CMJ assisted with sample collection and performed the lithium insertion, Raman spectroscopy and TEM measurements and participated in drafting the manuscript. All authors have approved the significance of the work, interpretation of results, and contents of the final manuscript.
